# The dominance of haptics over audition in controlling wrist velocity during striking movements

**DOI:** 10.1007/s00221-015-4529-9

**Published:** 2016-01-20

**Authors:** Yinan Cao, Bruno L. Giordano, Federico Avanzini, Stephen McAdams

**Affiliations:** Crossmodal Research Laboratory, Department of Experimental Psychology, University of Oxford, Oxford, UK; Department of Music Research, Centre for Interdisciplinary Research in Music, Media and Technology, McGill University, Montreal, QC Canada; Institute of Neuroscience and Psychology, University of Glasgow, Glasgow, Scotland, UK; Department of Information Engineering, University of Padova, Padua, Italy

**Keywords:** Striking objects, Surface stiffness, Crossmodal congruency, Motor control

## Abstract

Skilled interactions with sounding objects, such as drumming, rely on resolving the uncertainty in the acoustical and tactual feedback signals generated by vibrating objects. Uncertainty may arise from mis-estimation of the objects’ geometry-independent mechanical properties, such as surface stiffness. How multisensory information feeds back into the fine-tuning of sound-generating actions remains unexplored. Participants (percussionists, non-percussion musicians, or non-musicians) held a stylus and learned to control their wrist velocity while repeatedly striking a virtual sounding object whose surface stiffness was under computer control. Sensory feedback was manipulated by perturbing the surface stiffness specified by audition and haptics in a congruent or incongruent manner. The compensatory changes in striking velocity were measured as the motor effects of the sensory perturbations, and sensory dominance was quantified by the asymmetry of congruency effects across audition and haptics. A pronounced dominance of haptics over audition suggested a superior utility of somatosensation developed through long-term experience with object exploration. Large interindividual differences in the motor effects of haptic perturbation potentially arose from a differential reliance on the type of tactual prediction error for which participants tend to compensate: vibrotactile force versus object deformation. Musical experience did not have much of an effect beyond a slightly greater reliance on object deformation in mallet percussionists. The bias toward haptics in the presence of crossmodal perturbations was greater when participants appeared to rely on object deformation feedback, suggesting a weaker association between haptically sensed object deformation and the acoustical structure of concomitant sound during everyday experience of actions upon objects.

## Introduction

Sound is known to influence properties of tactile sensations such as the perceived frequency content of a vibration at the skin (Gescheider 1974; Guest et al. 2002) or the quantity of tactile taps (Bresciani et al. 2005). Furthermore, haptic information appears to dominate auditory cues across a broad range of audio–haptic processes (Lederman 1979; Caclin et al. 2002; Lederman et al. 2002; Lederman and Klatzky 2004; Soto-Faraco et al. 2004a; Giordano et al. 2012). This dominance echoes the “modality appropriateness” principle (Welch and Warren 1980) according to which sensory feedback signals are weighted based on long-term experience in proportion to the perceptual performance they afford. However, the majority of previous empirical evidence for the mechanisms of multisensory processing has been collected by focusing on purely perceptual situations in which multiple sources of information are combined to estimate sensory attributes (e.g., object size or hardness), hindering the understanding of how they are processed and weighted to control skilled motor performance.

Notable exceptions to this trend are studies that have examined the effect of integrating multiple sources of non-auditory feedback (e.g., visual and proprioceptive signals) on the early planning (Sober and Sabes 2003) and online correction of limb movements (Saunders and Knill 2004; Ronsse et al. 2009) in the absence of contact with external objects. However, we make frequent direct or tool-based contacts with objects when carrying out activities such as hitting a tennis ball with a racquet or playing a percussion instrument. In such cases, the task-relevant sensory information that can be transformed into goal-directed motor commands (Pouget and Snyder 2000) will include the somatosensory and auditory signals encoding the mechanical contact states. Maintaining dexterous strike-based interactions with sounding objects (e.g., drumming) requires fast and efficient updating of the kinematic state (e.g., wrist movement velocity) to counteract any sensory mismatch such as a mis-expectation of the impact properties based on the surface stiffness of the object.^1^ Sensory prediction errors (Miall and Wolpert 1996; Shadmehr et al. 2010) occur when there is a discrepancy between the predicted and the observed sensory consequences of a motor command. The prediction errors may be attributable to the alteration of multiple tactual cues (cutaneous and kinesthetic) that correlate with object surface stiffness (LaMotte 2000), and may naturally come from other sensory modalities apart from haptics, particularly audition (e.g., impact sounds). It is not clear by which mechanisms the integrative processing of impact-generated auditory and haptic information guides the control of striking kinematics during the sonic interaction with objects. We investigated this issue by measuring compensatory striking kinematics in a virtual-reality setup that allowed for the alteration of both haptic and auditory feedback as sensory consequences of striking events.

Expert percussionists have demonstrated superior precision compared to musically untrained individuals in both motor (Fujii et al. 2009) and perceptual tasks (Lutfi et al. 2011; Cicchini et al. 2012), indicating experience-related shaping of sensorimotor skills. It remains unclear, however, whether task-specific expertise can be a factor influencing the planning of compensatory motor responses based on crossmodal prediction errors. Musical training appears to enhance the sensitivity to audio–tactile conflicts in a detection task, supporting the predictive coding account of perception (Kuchenbuch et al. 2014). Motor-based multisensory processes, by contrast, have been robustly identified as involving a generalized state inference strategy that aims for a statistical optimization in terms of the lowest possible motor uncertainty independently of prior experience with the task (Ronsse et al. 2009). To evaluate whether or not experiential factors play a role in modulating motor-based multisensory processes, the present study included a group of professional percussionists as participants as well as a group of non-percussionist musicians and a group that was musically naïve.

Here participants learned to maintain the target velocity of a hand-held stylus while repeatedly striking an audio–haptic virtual object. With this setup, sensory feedback of the impact properties (acoustical and tactual) was experimentally determined not only by an intrinsic parameter—the surface stiffness of the audio–haptic object—but also by an extrinsic input—the striking velocity of the user. A surreptitious perturbation to the audio–haptic stiffness of the struck object would thus confuse the estimate of the source of prediction errors from impact properties (“limb or object?”), which would drive motor corrections. We quantified individual differences in the magnitude and direction of compensatory motor responses to different types of sensory error by manipulating the acoustical and haptic feedback in isolation. We furthermore addressed the issue of modality dominance by changing the auditory and haptic stiffnesses in combination, either incongruently (e.g., an increase in auditory stiffness heard in sound accompanied by a decrease in haptic stiffness felt by touch) or congruently. Sensory dominance would be characterized by a potential asymmetry of crossmodal congruency effects across audition and haptics (Driver and Spence 2004).

## Methods

### Participants

Forty-two right-handed participants (26 females; mean age = 22.5 years, SD = 4) were included in the study. They were divided into three groups of 14: non-musicians (very limited prior musical training, *M* = 0.9 years, SD = 1.4), non-percussionist musicians (NP-musicians, hereafter; at least four years of musical training, but not in percussion, *M* = 12.6 years, SD = 5.1), and percussionists (at least five years of professional training in percussion, *M* = 11.6 years, SD = 3.7; currently performing or practicing). All participants had normal hearing (Martin and Champlin 2000; ISO 2004), did not report any motor or haptic deficits, and were naïve with respect to the experimental goals. The protocol was certified by the McGill Review Ethics Board (Certificate 67-0905), and all participants gave written informed consent prior to the experiment.

### Apparatus

**Fig. 1:**
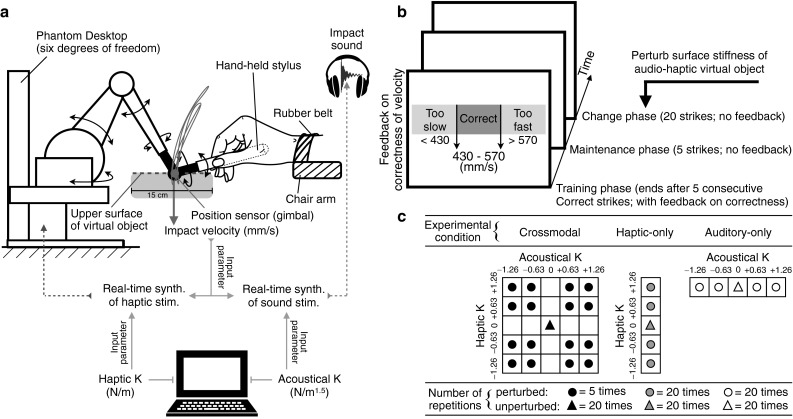
**a** Experimental apparatus. **b** Time course of one trial, comprising “training,” “maintenance,” and “change” phases. On the training phase, on-screen feedback is displayed immediately after a stroke to indicate whether the striking velocity at impact is below (“Too slow”), within (“Correct”), or above (“Too fast”) the target range. **c** Experimental conditions. *Circles* and *triangles* indicate the change-phase stiffness coefficient *K* values of the virtual object presented during the perturbed and unperturbed trials, respectively. *K* values are log-transformed and standardized for illustrative purposes. See “Stimuli” section for the real values of *K* used in the experiment

The task was performed inside an IAC model 120act-3 double-walled audiometric booth (IAC Acoustics, Bronx, NY). While seated in a chair, each participant was instructed to hold, with the dominant hand (right), the stylus of the Phantom Desktop™ (Geomagic Solutions, Morrisvelle, NC), a six-degrees-of-freedom robotic device used to deliver the haptic stimuli (Fig. 1a). The position signal of the tip of the stylus was measured at 1 kHz by optical encoders at the joints of the linkage structure connected to the stylus. Velocity was computed from position data using numerical differentiation along the vertical axis. This haptic device was interfaced with a real-time Pd (version 0.42.5)^2^ program for the synthesis of impact sounds, and with a custom C++ program for the control of haptic feedback (see “Stimuli” section below). Sound stimuli were amplified with a Grace Design m904 stereo monitor controller (Grace Digital Audio, San Diego, CA) connected to the optical port of a Windows workstation used to control the experimental variables, and were presented binaurally through Sennheiser HD280 headphones (Sennheiser Electronics GmbH, Wedemark, Germany). Verbal instructions about the task and visual feedback on the appropriateness of the participants’ performance were presented via the PC monitor (for details, see “Procedure” section, below).

The haptic device was stabilized on a table. The participant’s right forearm (except the hand and wrist) rested on the chair’s right arm, which was of the same height (15 cm above the table) as the upper surface of the virtual object (see “Stimuli” section). The right wrist was prone and lightly restrained with a rubber belt to the chair’s right arm so that only wrist flexion and extension could be easily used to displace the stylus. The joint angle between the upper and lower arm was adjusted to be approximately 90°. The participants’ hand, wrist, and forearm, as well as the stylus, were hidden from view.

### Stimuli

Auditory stimuli were synthesized impact sounds. Haptic stimuli were generated to simulate the sensory feedback of stylus-based strikes upon a static sounding object (Avanzini and Crosato 2006; Itkowitz et al. 2005; Fig. 1a). To impose perturbing effects on the struck object, we manipulated its mechanical parameter—surface stiffness coefficient *K* (hereafter surface stiffness will be referred to simply as stiffness). From the haptic standpoint, *K* establishes the relation between the object’s degree of deformation (surface indentation) and the perceived resistive force. From the auditory standpoint, higher values of *K* generate an increase in the subjective hardness of the struck virtual object perceived from the synthesized impact sounds (Giordano et al. 2010). Communication latency resulting from the sound synthesis was on the order of a few milliseconds, which is well below the typical experimental estimates for the temporal window of auditory–tactile integration (Bresciani et al. 2005). A previous study by Avanzini and Crosato (2006) using the same experimental setup reported that no participant perceived a noticeable intermodal latency.

#### Acoustical stimuli

**Fig. 2:**
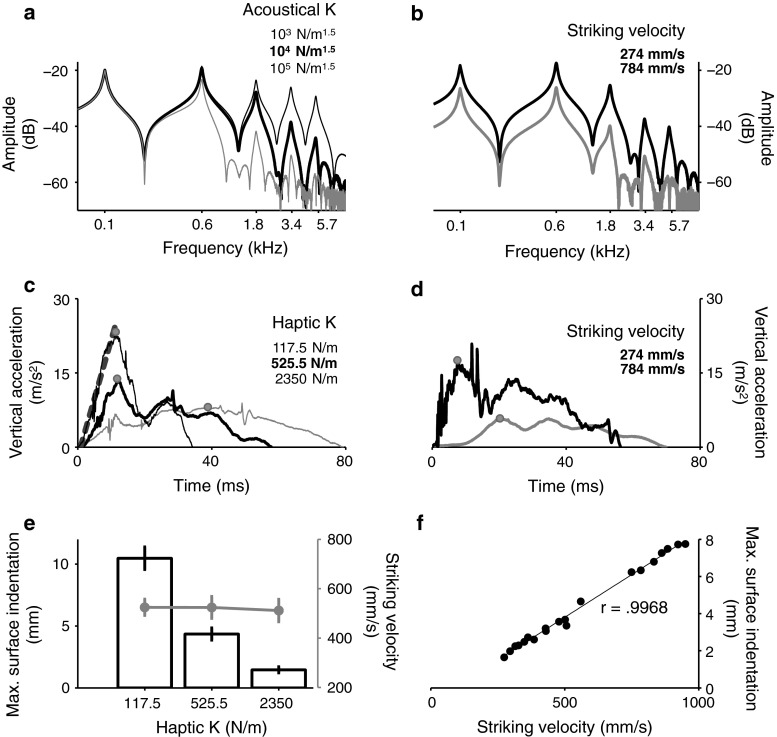
Properties of audio–haptic stimuli as a function of stiffness coefficient *K* and striking velocity at impact. **a**, **b** Spectra of impact sound as a function of either acoustical *K* (**a** synthesized with constant impact velocity of 500 mm/s) or striking velocity (**b** acoustical *K* = $10^{4}$ N/m$^{1.5}$). Vibrational modes with periods longer than contact duration fail to be excited efficiently; either larger acoustical *K* or higher striking velocity leads to shorter contact duration. **c**, **d** Resistive force, reflected by the vertical acceleration (stylus–object contact starts at $t=0$) of the stylus as a function of either haptic *K* (**c** equivalent striking velocities, *M* = 491, SD = 16 mm/s) or striking velocity (**d** haptic *K* = 525.5 N/m). The harder the object is, the greater are the magnitude and rate of resistive force produced by the object against the stylus during tapping. *Green dots* indicate force peaks. An example *red dashed line* (linear least-squares fit) shows the increment rate of the resistive force measured on the virtual object with the highest haptic *K* of 2350 N/m. **e**, **f** Deformation (indentation) cue, the maximal vertical displacement (*black bars*) of the stylus relative to the virtual object surface decreases with increasing haptic *K* (**e** equivalent striking velocities across haptic *K*s, one-way ANOVA $p>.96$), but increases with increasing striking velocity (**f** haptic *K* = 525.5 N/m). In **e**, *gray dots* represent the mean stylus velocities used to strike the three different haptic surfaces. *Error bars* indicate ±1 SEM (*N* = 20 strikes). Note that the indentation of the object by the stylus reaches a maximum simultaneously with the peak resistive force in **c** and **d** (color figure online)

Impact sounds (44.1 kHz, 16-bit resolution; peak intensity = 75 dB SPL) were synthesized using a real-time physically inspired model of an ideal struck bar with five independent vibrational modes and an internal dissipation (Avanzini and Crosato 2006). The resonant frequency of the lowest vibrational mode, *F*, was set to 100 Hz. Higher-order modal frequencies were tuned according to the most prominent resonances of a bar clamped at one end and free at the other (Fletcher and Rossing 1991), i.e., they were multiples of *F* by {6.26, 17.54, 34.37, 56.82}. The properties of the synthesized acoustical signal were determined in real time by two input parameters—acoustical stiffness coefficient (acoustical *K*; see Fig. 2a) and striking velocity at impact (Fig. 2b). Both the acoustical *K* and the impact velocity are inversely correlated with the contact duration (Chaigne and Doutaut 1997) in the model, which consequently affects the loudness and brightness of the attack portion of the impact sound (Giordano et al. 2010). A decrease in the contact duration would result in a more efficient excitation of the high-frequency vibrational modes of the sounding object and, consequently, an increase in the high-frequency energy of the radiated sound. In this study, acoustical *K* could vary across five log-spaced levels {$10^{3},\,10^{3.5},\,10^{4},\,10^{4.5},\,10^{5}$} N/m$^{1.5}$ centered around the baseline level of $10^{4}$ N/m$^{1.5}$.

#### Haptic stimuli

Impact-related touch feedback was simulated using a dissipative contact model when the head (gimbal) of the hand-held stylus reached the surface of the virtual object ($15 \times 15$ cm surface area; Fig. 1a). A reaction torque generated by the motors inside the device was delivered to the linkage structures connected to the stylus, simulating a vertical resistive force proportional to the instantaneous normal displacement of the stylus gimbal relative to the virtual object’s upper surface. The force was determined by a linear combination of the gimbal’s vertical displacement and velocity, weighted by a haptic stiffness coefficient (haptic *K*) and a dissipative (damping) coefficient, respectively. Both the haptic *K* and the striking velocity at impact could modify the haptic feedback, but differentially for the force-related (Fig. 2c, d) and deformation-related signals (Fig. 2e, f). Haptic feedback was programmed with the OpenHaptics™ Toolkit (Itkowitz et al. 2005). In this study, haptic *K* could assume one of five log-spaced values {117.5, 248.5, 525.5 (baseline), 1111.2, 2350.0} N/m. The largest stiffness coefficient here corresponds to the maximum stiffness (along the vertical direction) that can be achieved by the Phantom Desktop.

### Procedure

A schematic that depicts the experimental design and sequence is presented in Fig. 1. Each trial was divided into three consecutive phases (Fig. 1b). During an initial training phase, participants learned to constrain the striking velocities of their downswings within a target range (430–570 mm/s). They received on-screen feedback on a strike-by-strike basis: “Too slow,” “Correct,” or “Too fast” was displayed immediately after a stroke if the velocity at impact was below, within, or above that range, respectively. This phase ended when a participant had produced five consecutive strikes with the “correct” velocities. During a subsequent maintenance phase, participants continued striking with the same trained target velocity in the absence of feedback on correctness. This phase ended after five strikes independently of whether they were within or outside the target velocity range. The stiffness coefficient *K* of the virtual object was fixed to the baseline value across these two phases. At the beginning of a final change phase (without feedback on correctness), the values of the acoustical and/or haptic *K* either remained the same or were suddenly altered, alone or in combination, depending on the experimental condition. Participants were asked to continue striking with the same trained velocity and to ignore any changes in the properties of the object. This last phase ended after 20 strikes. Participants were required to strike with a tempo of their choice, provided that they kept it unchanged throughout an entire trial and did not exceed three strikes/second.

We investigated three conditions (Fig. 1c). In the haptic-only condition (100 trials), participants were presented with the virtual haptic object and with a continuous white-noise auditory masker presented at 75 dB SPL. In the auditory-only condition (100 trials), when the stylus reached the contact position, participants were presented only with the synthesized impact sound but no impact-related haptic feedback. During the audio–haptic (crossmodal) condition (100 trials), both the impact-related haptic and acoustical feedback were available. During the change phase of a crossmodal trial, the acoustical and haptic *K* of the virtual object could assume either the baseline value (unperturbed trial) or any of the 16 factorial combinations of the non-baseline values (perturbed trial). Crossmodal perturbations also depended on audio–haptic congruency. In the congruent condition, both the acoustical and haptic *K* either increased or decreased together relative to the baseline level. In the incongruent condition, the acoustical and haptic *K* changed in opposite directions (acoustical *K* increased, whereas haptic *K* decreased, or vice versa).

Each participant completed 20 blocks of 15 trials for a total of 300 trials in three sessions of approximately 1.5 h each on different days. Each block was subdivided into five subblocks during which participants were presented with three trials of different conditions (random order of conditions within each subblock). The 15 trials per block comprised five auditory-only trials (different change-phase acoustical *K* values), five haptic-only trials (different change-phase haptic *K* values), and five audio–haptic trials (one unperturbed trial and equal numbers of congruent and incongruent trials). Throughout all the audio–haptic trials, each of the possible combinations of the change-phase non-baseline haptic and acoustical *K* values was repeated five times, whereas the baseline value of the haptic–acoustical *K* was repeated 20 times. This design thus balanced each of the five *K* levels of the two dimensions (acoustical vs. haptic) with an identical number of trials for the modality-specific and crossmodal conditions, e.g., the haptic *K* of 117.5 N/m occurred equivalently 20 times for the haptic-only condition and the audio–haptic condition.

## Results

Data were analyzed with two linear mixed-effects models (LMM) fitted using the SAS^®^ PROC MIXED routine (West et al. 2006). The mixed models were used primarily because, in contradistinction to traditional models such as repeated-measures ANOVA, they make it possible to estimate directly the participant-specific regression coefficients for the main independent variables. The first analysis concerned the extent to which the modality-specific stiffness perturbations gave rise to motor compensations. The second analysis assessed the effects of crossmodal congruency on the interaction patterns for the motor effects of simultaneously available haptic and acoustical perturbations. The LMMs included both fixed effects (constant parameters) and random effects (assumed to follow normal distributions) measuring the average behavioral trend (mean linear motor effect of different levels of stiffness perturbation) and its interindividual variability within the population of interest, respectively. We adjusted *p* values with the Bonferroni correction for multiple post hoc comparisons.

### LMM 1: Stiffness perturbations prompted motor compensations in auditory-only and haptic-only conditions

The first LMM analyzed the effects of perturbing the virtual object’s stiffness coefficient *K* on the subsequent striking velocities in the two modality-specific conditions—auditory-only and haptic-only.

For each trial, we considered the average change-phase velocity across the last 19 strikes because the first strike of the change phase could not reflect the motor effect of the perturbed sensory feedback. These trial-specific motor measures were then collapsed into five means corresponding to the five different levels of the stiffness coefficient *K* per condition (see “Methods” section), yielding 10 data points (five for auditory-only; five for haptic-only) for each of the 42 participants.

**Fig. 3:**
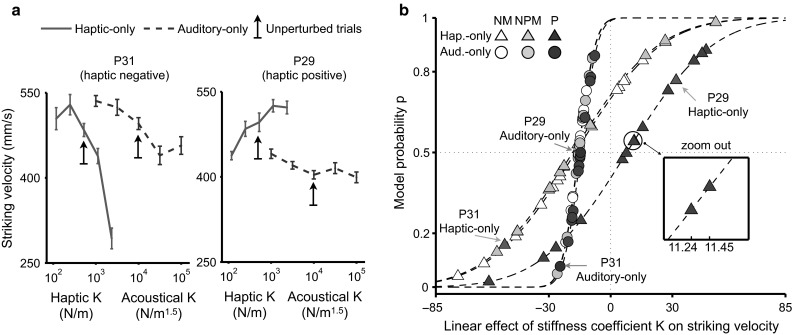
Effects of perturbed stiffness coefficient *K* on striking velocity in modality-specific conditions. **a** Typical participants who decrease (*left*; P31) and increase (P29) striking velocity for stiffer haptic surfaces. Mean change-phase striking velocity is plotted as a function of haptic and acoustical *K* in two different modality-specific conditions (*red* haptic-only; *blue* auditory-only). *Upward pointing arrows* indicate the data of unperturbed trials (constant *K*). *Error bars* represent ±1 SEM ($N=20$ trials). **b** LMM estimates of the participant-specific linear effects (fixed plus random) of the stiffness coefficient *K* on striking velocity. Fixed-effect estimates correspond to the *x*-axis values for which the Gaussian cumulative distribution functions (CDFs, *dashed curves*) reach the probability level of 0.5; random-effect estimates correspond to the variances of the CDFs. *Symbol color* and *shape* correspond to Expertise and Condition, respectively. Specific *K*-effect estimates for the typical participants in **a** are identified for illustrative purposes. *NM* non-musician, *NPM* non-percussionist musician, *P* percussionist (color figure online)

We examined the fixed effects of *K*, Condition (auditory-only vs. haptic-only), and musical Expertise, and of all the possible interactions between these factors. When fitting the models, log-transformed values of haptic and acoustical *K* were standardized (*z* scores), resulting in five linear-spaced levels from −1.26 to 1.26. Meanwhile, we included in this LMM significant random effects of both the participant-specific slope coefficients associated with *K* and the participant-specific intercepts [$\chi ^2(1,2)\,\ge\,15.1, \ p\mathrm {s}\,<\,.001$]. This LMM explained 73.2 % of the total variance (unadjusted $R^2$) in the striking velocity. The results of the LMM are illustrated by the normal cumulative distribution functions (CDFs, Fig. 3b), with the fixed-effect estimates corresponding to the abscissa values for which the CDFs reach the probability level of 0.5 and with the random-effect estimates corresponding to the variances of the CDFs.

Overall, the participant-specific slope coefficients of *K*, which represent the linear motor effects of stiffness perturbation, were significantly different from zero [$F(1,78)=51.1, \ p\,<\,.0001$]. The three-way *K* × Expertise × Condition interaction was not significant [$F(2,291)=2.84, \ p=.057$]. However, 10 of the 14 percussionists (eight of whom were mallet percussionists, see “Discussion” section) increased their striking velocity for higher values of haptic *K* (stiffer haptic surface; rightmost CDF for the effects of haptic *K* in Fig. 3b), whereas the percentage of the non-percussionists who tended to strike with lower velocity to compensate for higher values of the same variable (leftmost CDF for the effects of haptic *K* in Fig. 3b) was above chance (68 %). The mean fixed effect of haptic *K* was estimated to be −17.2 for non-musicians and −18.5 for NP-musicians, indicating indistinguishable patterns of motor compensation among these non-percussionists. To the contrary, percussionists had a positive (7.87) average estimate for the same fixed effect (see the *x*-axis values for which the dashed CDFs reach the model probability level of 0.5 in Fig. 3b). Nonetheless, the amount of previous experience with percussion performance was not a robust predictor for the large interindividual differences in the direction of compensatory motor responses to the haptic perturbations as represented by the sign of the linear effect of haptic *K* on striking velocity: The effect of musicianship could not explain why nine of the 28 non-percussionists were estimated to have positive linear effects for the haptic *K* as well (i.e., increasing striking velocity for higher values of haptic *K*).

To the contrary, the motor effect of acoustical *K* was consistent across the three groups [$F(2,291)\,<\,1$ for the post hoc between-group contrast within the auditory-only condition]. That is, an increase in acoustical *K* led to a decrease in striking velocity in all participants (average fixed-effect estimate of acoustical *K* was −15.2, ${\rm SD}\,=\,.58$). Note that the three CDFs for acoustical *K* (filled circles) were in close proximity in Fig. 3b.

Participants produced, on average, higher striking velocities in the haptic-only condition than in the auditory-only condition [$F(1,291)=23.8, \ p<.0001$]. This result was due to the fact that the change-phase striking velocities of the unperturbed haptic-only trials ($M=518$ mm/s) were significantly higher than those of the unperturbed auditory-only trials ($M=485$ mm/s), [$t(41)=4.51, \ p < .0001$]. The change-phase striking velocity of the unperturbed trials was not of primary interest in this study. A rank correlation analysis revealed that the absolute value of the participant-specific linear motor effect of different levels of stiffness perturbation was independent of the average striking velocity of the unperturbed trials for both modality-specific conditions [$|\rho (40)| \,\le\,.13, p\mathrm{s}\,\ge\,.12$]. None of the fixed effects of Condition × Expertise, *K* × Condition, *K* × Expertise, and Expertise per se, were significant [$F\,\le\,1.73, \ p\mathrm {s}\,\ge\,.18$].

In summary, this LMM revealed negative estimates for the motor effect of acoustical perturbation across all participants. The results indicate that unexpectedly higher acoustical *K*s (and thus auditory error signals in terms of unexpected increases in loudness and brightness) consistently led to a motor compensation that was characteristic of lower striking velocities. Meanwhile, this analysis revealed 19 positive and 23 negative participant-specific estimates for the motor effect of haptic perturbation. In other words, these two *classes* of individuals compensated for an increase in the stiffness of the haptic object by increasing ($N=19$) and decreasing ($N=23$) the striking velocity, respectively. From now on, we refer to these classes of individuals as *haptic positive* and *haptic negative*, respectively. The results reported here suggest a preferential reliance on different tactual error signals for the control of wrist velocity among different participants, although those error signals coexisted during the haptic perturbations. Specifically, increases in the struck virtual object’s stiffness would lead to decreases in the magnitude of a source of haptic deformation feedback, the object’s maximal surface indentation by the stylus (Fig. 2e). To compensate for the unexpectedly altered deformation feedback, the haptic-positive participants increased the striking velocity to restore the amount of indentation of the object by the stylus (Fig. 2f). To the contrary, the harder the object is, the greater are the magnitude and rate of the resistive force produced by the struck object against the stylus during tapping (Fig. 2c). To compensate for the suddenly increased values of these force-related cues, the haptic-negative participants decreased the striking velocities (Fig. 2d: slower velocities result in decreased values of the force-related cues given a constant haptic stiffness). In the LMM reported next, the analysis of the effects of crossmodal congruency will allow us to further characterize the interaction patterns for the motor effects of simultaneously available haptic and auditory error signals. We will be particularly interested in examining the extent to which the two different classes of participants characterized by the opposite directions of motor compensation for the same haptic perturbation differ in their strategies for weighting the error signals from haptics and audition for motor control.

### LMM 2: crossmodal congruency effects were asymmetrical between audition and haptics

The second LMM analyzed the effects of perturbing the virtual object’s stiffness on the average change-phase striking velocities (last 19 strikes) from the crossmodal perturbed trials. We adopted the idea of contrasting the behavioral effects of crossmodally congruent with incongruent conditions to investigate sensory dominance (Soto-Faraco et al. 2004b). With our experimental paradigm in particular, we concluded for the dominance of a sensory modality (e.g., haptics) if the motor effects induced by a manipulation of the modality-specific stiffness were more robust against the manipulation of crossmodal congruency than was the case for the non-dominant modality (e.g., audition).

**Fig. 4:**
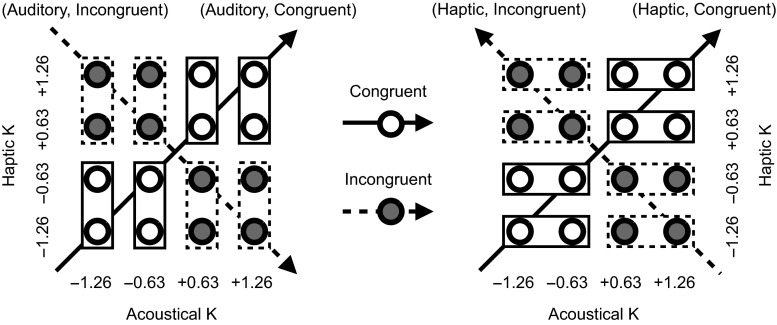
Collapsing data into four vectors for the second LMM according to Modality × Congruency. Stiffness coefficient *K*s are shown in log-transformed and standardized values. The *rectangle frames* denote “averaging” operators, with the *solid* and the *dashed frames* being averaged for the congruent and the incongruent audio–haptic conditions, respectively. *Arrow* directions show increasing *K* associated with the relevant modality

For each participant, the mean striking velocity was taken for each of the 16 possible combinations of the non-baseline haptic and acoustical *K* levels. Critical to this LMM, these 16 measures were collapsed according to a $2\times 2$ factorial contrast between sensory modality and crossmodal congruency, yielding four independent data vectors (congruent vs. incongruent, per modality) of four data points each (see Fig. 4). That is, we determined the motor effect of perturbation in one modality while averaging the change-phase striking velocities at different levels of perturbation in the other modality. For example, the motor effect of the four different levels of acoustical perturbation in the congruent condition (“Auditory, Congruent” in Fig. 4) was determined by averaging the change-phase striking velocities at the two levels of congruent haptic *K* associated with each of the four non-baseline acoustical *K* levels.

We examined in this model not only the fixed factors of the stiffness coefficient *K*, Modality (auditory vs. haptic), and Congruency, but also the participant-level covariates of Class (haptic positive vs. haptic negative) and musical Expertise (non-musician vs. NP-musician vs. percussionist), as well as the possible interactions among these factors. Meanwhile, we kept in the LMM significant random effects of both the participant-specific slope coefficients associated with *K* and the participant-specific intercepts [$\chi ^2(1,2)\,\ge\,61.7, \ p\mathrm {s}\,<\,.0001$].

This model explained 87.6 % of the total variance in the input data. It revealed significant fixed effects of *K* [$F(1,80)=5.38,\ p=.023$], Congruency [$F(1,496)=4.02,\ p=.046$], *K* × Congruency [$F(1,496)=20.1,\ p\,<\,.0001$], and *K* × Modality [$F(1,80)=18.3,\ p\,<\,.0001$], but not of Modality itself [$F(1,80)\,<\,1$]. Critical to this analysis, there was a significant three-way *K* × Congruency × Modality interaction [$F(1,496)=29.7,\ p\,<\,.0001$]. A pairwise between-modality contrast (haptic minus auditory) for the congruency-related absolute changes in the estimated linear effects of *K* revealed that the altered haptic feedback dominated the altered acoustical feedback in determining the motor compensation [$t(496)=-4.90,\ p\,<\,.0001$]. In particular, the population-averaged estimate for the motor effect of haptic *K* was shifted to a lesser degree by the crossmodal congruency (an asymmetry of the congruency effects in favor of haptics), compared with that of acoustical *K*. The mean congruency-related absolute changes in the estimated linear effects of haptic and acoustical *K* on striking velocity were 20.2 and 39.6, respectively.

**Fig. 5:**
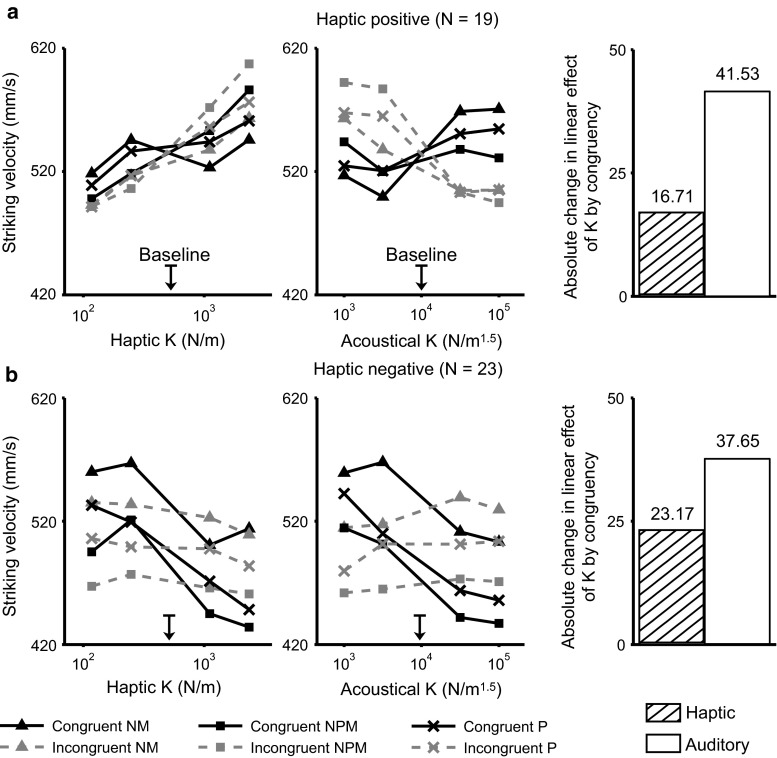
On each crossmodal perturbed trial, both the haptic and acoustical *K* were manipulated (congruent = both increased or decreased relative to the baseline during the change phase of each trial; incongruent = one increased while the other decreased). The *leftmost* and *middle subplots* show the across-participant means of change-phase striking velocity as a function of haptic or acoustical stiffness, averaged across levels of the stiffness values for the other modality. The *rightmost subplots* show the absolute change in the linear effect of acoustical *K* and haptic *K* on striking velocity by crossmodal congruency. The *bar height* represents the susceptibility of a sensory modality to the influence of an incongruent crossmodal perturbation. *Top* (**a**) and *bottom* (**b**) panels report results for the haptic-positive and haptic-negative participants, respectively. *NM* non-musician, *NPM* NP-musician, *P* percussionist

Furthermore, the between-modality difference (haptic minus auditory) for the congruency-related absolute changes in the linear effects of *K* was greater for the haptic-positive individuals than for the haptic-negative individuals [$t(496)= 2.43,\ p\,=\,.015$]. This result indicates that the asymmetry of the crossmodal congruency effects between audition and haptics was more pronounced for the haptic-positive individuals than for the haptic-negative individuals. We used the population-averaged estimate for the congruency-related absolute change in the linear effect of *K* as a measure for deriving the relative weights of the two sensory modalities. For example, the relative weight of haptic feedback for the haptic-positive participants was $41.53/(41.53+16.71)\,=\,0.71$ (Fig. 5a, rightmost subplot). Overall, the haptic-positive individuals gave greater weight to the haptic inputs (71 %) than to the auditory inputs (29 %), which underpinned their stronger response bias toward haptics when compared with the relative weighting of haptics and audition (62 vs. 38 %, respectively) in the haptic-negative individuals (Fig. 5b, rightmost subplot).

In addition, the *K* × Class effect was significant [$F(1,496)=47.9,\ p\,<\,.0001$], with the average linear effect of *K* for the haptic-negative participants being estimated to be 39.5 (${\rm SE} = 6.58$) units less than that of the haptic-positive participants. The Class effect per se was significant [$F(1,496)=10.3,\ p=.0014$], with the mean change-phase striking velocity of the haptic-negative participants being 43.4 (${\rm SE}\,=\,22.8$) mm/s lower than that of the haptic-positive participants. The LMM also revealed significant fixed effects of *K* × Modality × Class and *K* × Congruency × Class interactions [$F(1,496)\ge 34.8,\ p\mathrm {s}\,<\,.0001$]. To more closely inspect these effects, we compared separately for the haptic-positive and haptic-negative individuals the motor effects of *K* in each modality and congruency condition.

Haptic-positive individuals tended to increase their striking velocity for an unexpectedly stiffer haptic surface that was presented alone (haptic-only), which we hypothesize shows a preference for deformation feedback. Their results were characterized by the same motor compensation strategy when receiving the acoustical and haptic perturbations simultaneously, independently of crossmodal congruency (Fig. 5a, leftmost subplot). In particular, compared to the haptic-only perturbation, the estimated linear effect of *K* was also positive (regardless of congruency). The positive motor effect of haptic *K* was larger for the incongruent (32.9) than for the congruent (16.2) trials, indicating that a stiffer haptic object combined with a softer impact sound (or vice versa) could induce an enhancement in the magnitude of motor compensation. The motor effect of acoustical *K*, which was originally negative (e.g., striking velocity was decreased for "harder" impact sounds) when the acoustical feedback was presented alone, turned out to be reversed by the strong positive motor effect of haptic *K* in the congruent audio–haptic condition (Fig. 5a, middle subplot).

Haptic-negative participants may have relied preferentially on force-related feedback, given that they compensated for a stiffer haptic surface by decreasing the striking velocity in the haptic-only condition. Adding an incongruent yet task-relevant acoustical perturbation appeared to affect more strongly their compensation for the altered haptic feedback in the crossmodal context, compared with the haptic-positive participants mentioned above (Fig. 5b, leftmost subplot). The motor effect of haptic *K* estimated in the LMM for the incongruent crossmodal trials, albeit still negative, was quite small on average (−6.52) if compared with that for the congruent trials (−29.7). As shown in Fig. 5b (middle subplot), the haptic dominance was still pronounced here, as revealed by a positive estimate (6.14) for the motor effect of acoustical *K* in the incongruent crossmodal condition (the preceding LMM revealed negative motor effects of perturbing the same variable during the auditory-only trials).

Finally, the effect of musical Expertise was not significant [$F(2,496)=1.50,\ p=.22$], but it was modulated by the effect of Class [$F(2,496)=3.16,\ p=.043$], with significantly lower striking velocity on average for the NP-musicians compared with the non-musicians, but only within the haptic-negative class of participants [$t(496)=-3.78,\,p=.0012$; $|t(496)|\,\le\,1.71,\,p\,\ge\,.53$ for the other contrasts between expertise-related groups]. Notably, any other expertise-relevant effects failed to reach statistical significance [$F(2,496)\,<\,1$], indicating that the above-mentioned motor effect of *K* and its interactions with other factors (i.e., Modality, Congruency, Class, or interactions between these) were generalizable across the three expertise-related groups. We also fitted a reduced model omitting only the Class effect from the model presented above while preserving all the other effects and the variance–covariance structures. In this reduced model, musical Expertise failed to modulate any *K*-related fixed effect, i.e., the effects of *K*, *K* × Modality, *K* × Congruency or *K* × Modality × Congruency [$F\,\le\,1.93, \ p\mathrm {s}\,\ge\,.15$]. Taken together, these results demonstrated that the participant-level covariate of Class was more capable of predicting the behavioral variance in the weighting of auditory and haptic feedback for motor control than was the amount of previous experience with percussion performance.

To summarize the congruency analysis, we found that the acoustical feedback was processed differently depending on whether it was congruent with the altered information from haptics or not. The altered haptic feedback dominated the altered acoustical feedback in determining the magnitude and direction of the compensatory motor responses in cases of crossmodal perturbation. These congruency effects depended on the factor of Class: Stronger response bias toward haptics was found in the haptic-positive individuals who appeared to show greater sensitivity to the altered deformation feedback, compared with the haptic-negative individuals. Further, this dependency has the same pattern for the three expertise-related groups. Essentially, musicianship did not have an influence on the weighting of auditory and haptic feedback for motor control beyond what could be accounted for by the choice of which tactual error signal, deformation or force, was the most relevant to the motor task.

## Discussion

In this study, the impact-related haptic feedback (surface indentation and resistive force) and auditory feedback (sound loudness and brightness) were altered alone or simultaneously as participants repeatedly struck a virtual sounding object. The compensatory changes in striking velocity were measured as the motor effect of the sensory perturbation. Overall, changes in the surface stiffness of the object in both the haptic and auditory modalities led to compensatory motor responses. However, an analysis of the effects of crossmodal congruency of stiffness change in the audio–haptic context revealed a robust dominance of haptics over audition in the control of striking velocity. Further, whereas the motor effects of an auditory perturbation were largely consistent across participants, those of a haptic perturbation varied widely, revealing distinct participant-specific compensatory strategies, i.e., a consistent increase versus a consistent decrease in striking velocity following an increase in haptic stiffness across trials. We argued that these opposite compensatory strategies resulted from a reliance on two different types of tactual error signals—surface deformation and vibrotactile force.

Interestingly, both haptic perturbation compensatory strategies were observed for participants in all musicianship groups, albeit with a slight, but non-robust, prevalence for a velocity increase with haptic stiffness and thus a potential reliance on surface deformation in percussionists. More importantly, we observed that within a crossmodal context, the weighting of auditory and haptic information for motor control was heavily dependent on whether participants focused on surface deformation or vibrotactile force, and was largely unaffected by the amount of previous experience with percussion performance per se. This finding echoes the absence of task-relevant expertise effects on the optimality-based prediction of sensory dominance across different learning stages for motor execution in a previous study that employed a bimanual coordination task involving cyclical wrist movements (Ronsse et al. 2009). Notably, these results do not necessarily contradict previous reports of experience-dependent shaping of an enhanced sensitivity to crossmodal incompatibility (Powers et al. 2009; Lee and Noppeney 2011; Kuchenbuch et al. 2014). Indeed, those studies did not examine action-based contexts and did not focus on the weighting of sensory feedback, but on the role of experience-dependent multisensory-coupling priors on the degree of sensory cue fusion (Ernst and Di Luca 2011).

Previous psychophysical evidence has shown dominance of haptic information over auditory information in perceptual tasks of estimating object textures (Lederman 1979; Lederman et al. 2002), judging apparent motion (Soto-Faraco et al. 2004a) or spatial localization (Caclin et al. 2002), or identifying materials (Giordano et al. 2012). Our results provide evidence of haptic dominance over audition in a sonic interaction scenario. In particular, the acoustically induced motor responses were modulated more strongly by a synchronized haptic perturbation than vice versa, as revealed by a marked asymmetry of the congruency effects across audition and haptics. The stronger weighting of haptics than audition might be attributable, at least in part, to a bias toward focusing on a sensory modality—haptics—that is more tightly linked to the exploration and exploitation of effector-related information during active sensorimotor activities. For instance, in our experiment, the striking velocity is simply the derivative of deformation with respect to time, and given a constant mass of the hand–stylus system, it is the integral of resistive force with respect to time. As such, focusing on tactual error signals would constitute a more efficient strategy for motor control because it would potentially require fewer computationally intensive sensorimotor transformations than attending to auditory error signals (Pouget and Snyder 2000). This superior modality-specific utility could also be learned developmentally through long-term tactual experience of object manipulations and explorations. For instance, the identification of aggregate materials (e.g., gravel of different sizes) through locomotor movements involved during walking appears to be dominated by the kinesthetic information that most promptly signals a potential postural instability (Giordano et al. 2012). We also noticed that the acoustical perturbation did lead to a small but observable effect on the haptically induced motor responses, drawing a parallel with analogous reciprocal audio–tactile interactions (albeit with an asymmetry in favor of touch) at perceptual levels of motion processing (Soto-Faraco et al. 2004a). This result contrasts with the highly imbalanced modality preference during speech sensorimotor control (Lametti et al. 2012), where the motor compensation for somatosensory perturbation (altered motion path of the jaw) is not affected by a simultaneous auditory perturbation (altered spectral parameters). It remains unknown whether such mixed results are due to the neurophysiological distinction between the two sensorimotor systems (limb specific vs. vocal specific) or are the results of a methodological incompatibility: We employed an ecologically relevant manipulation of crossmodal prediction errors (based on audio–haptic stiffness), whereas Lametti et al. (2012) manipulated the uncorrelated cues of movement path of the jaw and formant frequencies of the utterance.

Given the nature of our experimental manipulation of haptic feedback, an unexpected increase (decrease) in the virtual object’s haptic stiffness would result in a sudden decrease (increase) in the perceived magnitude of deformation (i.e., surface indentation) of the object by the stylus, yet a larger (smaller) magnitude and faster (slower) increment rate of the resistive force generated during the simulated mechanical contact.^3^ Striking an object with higher velocities (greater energy supplied to the vibrating system) would instead always generate increases in the values of various tactual cues (Fig. 2c–f). The resulting motor compensation for the altered deformation feedback versus force feedback should thus operate in opposite directions, making it possible to group participants based on whether they responded to either type of error signal consistently across experimental trials. Individual differences indeed emerged in the pattern of compensatory motor responses to the haptic perturbation, which could be most parsimoniously explained in terms of a preferential reliance upon different tactual error signals. Fifty-five percent of the participants (“haptic negative”) likely tended to compensate for the altered force cues (maximal resistive force and resistive force rate) by striking more softly (lower striking velocity) on an unexpectedly stiffer object. To the contrary, 45 % of the participants (“haptic positive”) appeared to compensate for the altered surface deformation feedback (maximal indentation) by striking harder on the stiffer object in order to indent its surface as deeply as had been experienced prior to the perturbation.

Although the exact origin of the observed reliance on tactual cues for motor control might open up an avenue that merits further experimentation, two plausible but not mutually exclusive explanations can be put forward. First, the individual differences in susceptibility to the perturbation of tactual signals may be partially explained by variability in the sensory acuity (Villacorta et al. 2007) for the changes in the different tactual signals that are transmitted via separate information-processing channels. These channels would represent distinct mechanoreceptor populations and afferent fibers for tactile versus kinesthetic sensations. Alternatively, these interindividual differences might originate from previous experience with tool-based interactions with objects. Notably, we found that 10 out of 14 percussionists—eight of them being mallet percussionists who specialized in xylophone, marimba, etc.—showed a preferential reliance on the deformation feedback. Increased sensitivity to a particular type of error signal during percussion may be shaped by many years of experience with striking objects of different stiffness with mallets that also vary in stiffness. To control a manipulative behavior on objects, most people potentially employ a mechanism (an internal model of the object properties, Flanagan and Wing 1997) that seems not to require special expertise: Grasp stability is achieved by adjusting the grip force in anticipation of the changes in resistive force generated by a tapped object (LaMotte 2000). Mallet percussionists, however, may have potentially developed a different strategy. They routinely relax the grip so as to allow the hand-held mallet to rotate freely around a fulcrum point (commonly between the index finger and thumb) in a vertical plane (Dahl 2006). They in fact make an effort to keep the wrist joint as compliant as possible (to prevent fatigue) during repeated percussive gestures, which is achieved through unique physiologically efficient reciprocal activities of the wrist muscles (Fujii et al. 2009). Monitoring the wrist-rotation trajectories (position-related tactual signals encoded via joint receptors and muscle afferents) rather than the grip force would possibly facilitate a mallet percussionist’s superior sensitivity to the nuances in the magnitude of object indentation by the stylus. Future studies could include “non-mallet” percussionists who have been trained most of the time to play with their bare hands and fingers (e.g., tabla and conga players) in order to examine the extent to which the observed cue reliance is associated with the specificity of mallet-based percussion training. Issues such as strict criteria for participant selection must be considered cautiously, because modern percussionists are usually known for their versatility across a broad range of percussion instruments.

One last important finding of this study is that within a crossmodal context, actively monitoring deformation rather than force feedback results in a greater disregard for the altered auditory feedback. Potential explanations for this result rely on the hypothesis that the causal association between haptic deformation signals, on the one hand, and auditory cues, on the other, is weaker than that observed between force signals and auditory cues. From the short-term perspective, it might instead be argued that repeated manipulations of the haptic device in this experiment may have given rise to participants’ awareness that the synthesized sounds fail to carry any information about the object’s deformation by the stylus, although they still convey spatial and temporal information about the contacts with the surface (DiFranco et al. 1997). We argue that long-term sensorimotor experience with the striking of sound-generating solid objects made of materials that range from easily deformable to rigid results in reliable associations between resistive forces and concomitant impact sounds, but not between deformation and impact sounds. Indeed, objects that generate higher resistive forces also produce louder impact sounds. And although deformable objects tend to produce softer sounds, non-deformable materials such as hard wood or steel produce a wide range of loudness levels due to variations in their intrinsic vibrational and damping properties when struck with the same velocity. For this reason, through long-term experience, we may potentially learn that deformation is less reliably associated with the structure of the concomitant sound stimulus than are force cues. Importantly, the interaction of crossmodal channels for information processing can show a tendency to break down if a process of causal inference judges that the different sensory signals originate from weakly correlated sources (Körding et al. 2007; Ernst and Di Luca 2011). As such, auditory cues are more likely to be ignored if a participant shows predominantly greater sensitivity to the deformation-related prediction errors.

## Footnotes

1 The degree of *surface stiffness* of an object is defined as the magnitude of resistive force caused by a unit measure of surface indentation by an external indentor (such as the finger or stylus) in contact with the object surface. Surface stiffness is one among various other material properties of solid objects toward which perceptual experiences are directed through “active touch” (Gibson 1962), such as surface roughness, stickiness, spatial density, and apparent temperature. The perceptual correlates of these properties have proven to be reliably orthogonal to those of geometric properties that are specific to particular objects (see taxonomy of object properties by Klatzky and Lederman 1993). As a geometry-independent material property in the tactual psychophysics literature, surface stiffness must be distinguished from the mechanical stiffness of a structure, whose value is largely affected by geometric factors (e.g., a kitchen steel scrubber deforms easily when squeezed because of its structural design and not because of the inherent surface stiffness of steel; Rivin 1999).

2 M. Puckette, PureData (Pd), http://www.puredata.org, Accessed 6 April 2014.

3 These tactual cues correlate with object stiffness during active tapping with a rigid hand-held stylus (LaMotte 2000).
